# The Association between Resting Heart Rate and Urinary Albumin/Creatinine Ratio in Middle-Aged and Elderly Chinese Population: A Cross-Sectional Study

**DOI:** 10.1155/2019/9718370

**Published:** 2019-08-25

**Authors:** Wenfeng Mao, Xinye Jin, Haibin Wang, Yingnan Ye, Linxi Zhang, Shi Gu, Jie Wang, Guang Ning, Yiming Mu

**Affiliations:** ^1^Department of Endocrinology, Chinese People's Liberation Army General Hospital, No. 28 Fuxing Road, Beijing 100853, China; ^2^School of Medicine, Nankai University, No. 94 Weijin Road, Tianjin 300071, China; ^3^Department of Endocrinology, Ruijin Hospital, Shanghai Jiaotong University School of Medicine, Shanghai 200025, China

## Abstract

**Objective:**

In general population, resting heart rate (RHR) is associated with cardiovascular disease. However, its relation to chronic kidney disease (CKD) is debated. We therefore investigated the relationship between RHR and urinary albumin/creatinine ratio (UACR, an indicator of early kidney injury) in general population at different levels of blood pressure and blood glucose.

**Methods:**

We screened out 32,885 subjects from the REACTION study after excluding the subjects with primary kidney disease, heart disease, tumor history, related drug application, and important data loss. The whole group was divided into four groups (Q1: RHR ≤ 71, Q2: 72 ≤ RHR ≤ 78, Q3: 79 ≤ RHR ≤ 86, and Q4: 87 ≤ RHR) according to the quartile of average resting heart rate. The renal function was evaluated by UACR (divided by quartiles of all data in the center to which the subject belonged). Ordinary logistic regression was carried out to explore the association between RHR and UACR at diverse blood pressure and blood glucose levels.

**Results:**

The subjects with higher RHR quartile tend to have a higher UACR, even multifactors were adjusted. After stratifying the subjects according to blood pressure and blood glucose, the positive relationship between RHR and UACR remained in the subjects with normal blood pressure and normal glucose tolerance, while in the hypertension (SBP ≥ 140 mmHg and/or DBP ≥ 90 mmHg) group and the diabetic mellitus (FPG ≥ 7.0 mmol/L and/or PPG ≥ 11.1 mmol/L) group, the relationship disappeared. In the subjects without hypertension, compared with the Q1 group, the UACR is significant higher in the Q3 group (OR: 1.11) and the Q4 group (OR: 1.22). In the subjects with normal glucose tolerance (NGT), compared with the Q1 group, the UACR is significantly higher in the Q3 group (OR: 1.13) and the Q4 group (OR: 1.19).

**Conclusions:**

The population with higher RHR tend to have a higher UACR in the normal blood pressure group and the normal glucose tolerance group.

## 1. Introduction

Resting heart rate (RHR) is a well-recognized marker of autonomic nervous system tone and has been found to be a significant correlate of blood pressure, increased body mass index, and metabolic disturbances in many studies; this association is particularly striking in patients with hypertension or diabetes [1]. Elevated resting heart rate has been demonstrated to be associated with type 2 diabetes [2], metabolic syndrome [3, 4], cardiovascular disease, and all-cause mortality [5, 6].

The kidney is one of the important target organs of metabolic diseases. Chronic kidney disease (CKD), primarily caused by hypertension and diabetes [7], which could exacerbate or usually recurs and leads to impairment of renal function, increased risk of cardiovascular disease, cognitive disorders, anemia, and abnormal bone metabolism with untimely diagnosis and treatment. Urinary microalbumin is one of the sensitive indicators reflecting early renal function injury [8]. Many studies have reported that albuminuria is not only an independent risk factor of cardiovascular and cerebral diseases but also a relevant factor for stroke and cardiac cerebrovascular death [9–11].

A raised RHR has been found to be an independent predictor for the prevalence of microalbuminuria in hypertensive patients with high cardiovascular risk [12]. And people with type 2 diabetes mellitus who have a higher resting heart rate experience a greater incidence of new-onset or progressive nephropathy and retinopathy [13]. It has been shown that antihypertensive therapies are able to reduce the incidence of microalbuminuria, with blockers of the renin-angiotensin system being particularly effective [14, 15]. Furthermore, it was shown that a pharmacologically induced reduction in HR resulted in improvements of endothelial function in mice [16] and thus has the potential to reduce new-onset microalbuminuria in humans. The mechanism by which RHR affects the occurrence of microalbuminuria in diabetic patients remains unclear; it is likely that a combination of factors contributes to its development. Increases in pulse waves, glomerular pressure, base membrane permeability, inflammatory effects, and proatherosclerotic activity have been postulated to be involved in the process [12, 13, 17].

An early subanalysis of the randomized olmesartan and diabetes microalbuminuria prevention (ROADMAP) study on patients with type 2 diabetes has shown that a lower RHR was associated with a reduced risk of developing microalbuminuria, indicating that a lower RHR is an independent predictor for a lower risk of new-onset microalbuminuria in type 2 diabetes [18]. Our study extended the subjects to the general population, trying to explore the association between RHR and urinary albumin/creatinine ratio (UACR) at diverse blood pressure and blood glucose levels in general population from 8 different regional community cohorts in China.

## 2. Methods

### 2.1. Study Population

A total of 53,639 participants from the Risk Evaluation of cAncers in Chinese diabeTic Individuals a lONgitudinal (REACTION) study gave written informed consent, and the protocols were reviewed and approved by the research ethics committee at each of the participating centers (Dalian 10,140, Lanzhou 10,026, Guangzhou 9743, Luzhou 8105, Shanghai 6821, Guangxi 5831, Zhengzhou 1978, Wuhan 995). After excluding the subjects with primary kidney disease, heart disease, tumor history, related drug application, and important data loss, 32,885 participants were investigated.

### 2.2. Data Collection

The baseline data were collected by trained health workers via a standardized questionnaire during interviews, including the general situation, the past medical history, the current medication situation, lifestyle, physical exercise, smoking and drinking habits, family history, and other basic information. All involved investigators have been formally trained. Participants were asked to take off shoes, hats, and coats before measurements. Waist circumference (WC) was measured at the horizontal level of the midpoint of the ligature between anterior superior spine and inferior margin of the twelfth rib. Hip circumference (HC) was defined as the horizontal of the most protruding part of the hip. Body mass index (BMI) = weight (kg)/[height(m)]^2^.

An automated electronic device (OMRON Model HEM-725 FUZZY, Omron Company, Dalian, China) was used to measure blood pressure and resting heart rate in the nondominant arm of seated participants three times consecutively at 1-minute intervals after a ≥5-minute rest. The three readings were averaged for analysis. The subjects were stratified based on RHR quartiles (Q1: RHR ≤ 71, Q2: 72 ≤ RHR ≤ 78, Q3: 79 ≤ RHR ≤ 86, and Q4: 87 ≤ RHR).

Urine samples were collected in the morning (midclean urine) for UACR measurements. Because the kits for measuring UACR in each center and the range of normal values are different, the value of UACR, divided by quartiles of all data in the center which the subject belonged to, was used to estimate albuminuria.

Blood samples were drawn in the morning after subjects had an 8-hour fasting the previous night. Participants without a history of diabetes underwent a 75 g oral glucose tolerance test, and their venous blood samples were drawn at 0 and 120 minutes. Biochemical index included triglyceride (TG), cholesterol (TC), low-density lipoprotein cholesterol (LDL-c), high-density lipoprotein cholesterol (HDL-c), creatinine (Cr), alanine transaminase (ALT), aspartate transaminase (AST), gamma-glutamyl transaminase (GGT), fasting plasma glucose (FPG), postprandial plasma glucose (PPG), glycosylated hemoglobin (HbA1c), and fasting blood insulin was measured by the glucose oxidase-peroxidase method.

The estimated glomerular filtration rate (eGFR) was expressed in mL/min per 1.73 m^2^ by the formula eGFR = 186 × [serum creatinine × 0.011]‐1.154 × [age]‐0.203 × [0.742 if female] × 1.233, where serum creatinine was expressed as *μ*mol/L and 1.233 was the adjusting coefficient for the Chinese population. This formula is according to the abbreviated Modification of Diet in Renal Disease (MDRD), which was recalibrated for Chinese population.

### 2.3. Blood Glucose (mmol/L) and Blood Pressure (mmHg)

Normal glucose tolerance (NGT) was defined as FPG < 6.1 and PPG < 7.8; impaired glucose regulation (IGR) was defined as 6.1 ≤ FPG < 7.0 and/or 7.8 ≤ PPG < 11.1; diabetes mellitus (DM) was defined as FPG ≥ 7.8 and/or PPG ≥ 11.1. Normal blood pressure (NBP) was defined as 90 ≤ systolic blood pressure (SBP) < 140 and 60 ≤ diastolic blood pressure (DBP) < 90; hypertension was defined as SBP ≥ 140 and/or DBP ≥ 90.

### 2.4. Statistical Analysis

The data was analyzed by SPSS version 20.0 for Windows (SPSS Inc., Chicago, IL, USA). The normality of distribution of continuous variables was tested by the one-sample Kolmogorov-Smirnov test. Continuous variables with normal distribution were presented as mean ± standard deviation (SD). The Mann-Whitney *U* test and the Kruskal-Wallis test were used, respectively, to compare the means of 2 and 3 or more groups of variables not normally distributed. The frequencies of categorical variables were compared using Pearson Chi-square or Fisher's exact test, when appropriate. A value of *P* < 0.05 was considered significant. Ordinary logistic regression was performed to explore the association between RHR and UACR.

## 3. Results

### 3.1. Clinical Characteristics of the Study Population

**Figure 1:**
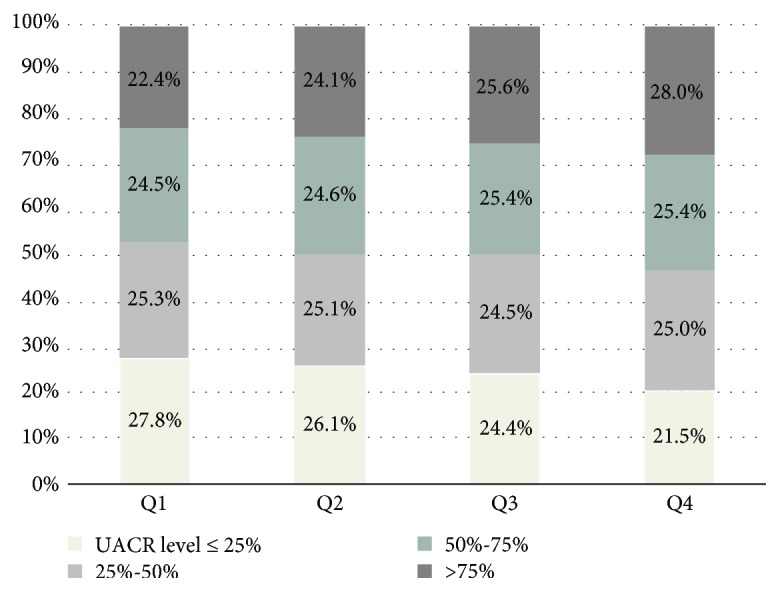
The percentile of the UACR level at diverse RHR.

A total of 32,885 participants, including 10,058 males and 22,827 females were accepted into the study. The general clinical characteristics of the study population are presented in Table 1. The subjects with the higher RHR were more likely to have higher DBP, FPG, PPG, HbA1c, insulin, TC, TG, LDL, ALT, AST, and GGT (*P* < 0.001). The means of age, HC, SBP, Cr, and eGFR have a significant difference between RHR quartiles. The percentile of UACR > 75% is higher in the higher RHR group as Figure 1 shows.

### 3.2. Statistical Analysis

Multiple factor analysis was carried out to screen out the risk factors related to UACR.  Table 2 shows that RHR, gender, blood pressure, and blood glucose are significantly associated with UACR (all *P* < 0.05). Ordinal logistic regression was performed to explore the association between UACR and RHR.  Table 3 presents the results. There was a positive relationship between RHR and UACR (all *P* < 0.05). After adjusted for gender, age, BMI, WC, smoking, and alcohol drinking, the risk of having UACR increased progressively across the lowest to the highest quartiles of RHR with ORs of 1.07 (95% CI 1.01-1.13), 1.18 (95% CI 1.12-1.25), and 1.33 (95% CI 1.26-1.41), respectively (all *P* < 0.05). When adjusted for all factors, the third quartile (OR: 1.13; 95% CI 1.07-1.20) and the fourth quartile (OR: 1.17; 95% CI 1.10-1.24) still tend to have a higher UACR.

### 3.3. Stratification according to Blood Pressure and Blood Glucose

The association between UACR and RHR at diverse blood pressure and blood glucose was analyzed by ordinal logistic regression. As Table 4 shows, in the nonhypertension group, the risk of higher UACR occurrence in the third quartile (OR: 1.11; 95% CI 1.04-1.18) and the fourth quartile (OR: 1.22; 95% CI 1.13-1.31) increased when compared to the first quartile; in the hypertension group, the positive relationship disappeared. As to the blood glucose level, in the normal glucose group, the risk of having UACR in the second quartile (OR: 1.07; 95% CI 1.00-1.15), third quartile (OR: 1.13; 95% CI 1.05-1.21), and fourth quartile (OR: 1.19; 95% CI 1.10-1.29) increased when compared to the first quartile, while in the impaired glucose regulation group and the diabetes mellitus group, we cannot observe the positive relationship.

## 4. Discussion

Chronic kidney disease has insidious onset and slow progression and is easy to be ignored. UACR is a sensitive indicator reflecting early renal function injury. Resting heart rate is a reliable indicator of sympathetic nervous system excitation, and epidemiological studies [19, 20] show that resting heart rate increases are closely related to the risk of hypertension, diabetes, and other diseases. The present study demonstrated the association between the RHR and the UACR level in general population from 8 regional centers of Chinese mainland. We found that there was a positive relationship between RHR and UACR. Compared to Q1 (RHR ≤ 71), the risk of UACR in the subjects belong to Q3 (79 ≤ RHR ≤ 86) and Q4 (87 ≤ RHR) increased, respectively, by 13% (OR: 1.13) and 17% (OR: 1.17), even after adjusting confounding factors such as gender, age, BMI, WC, smoking, alcohol drinking, blood pressure, blood glucose, ALT, AST, GGT, TC, TG, HDL-c, and LDL-c.

A previous study in Germany that included 4447 patients with type 2 diabetes showed that as the heart rate increases, the risk of microalbuminuria appearing increased [18]; this positive relationship is in accordance with our study. This indicates that lowering the resting heart rate may be a protective factor against microalbuminuria. However, the difference is that the study population in Germany is limited to patients with type 2 diabetes. In our study, we excluded the diagnosed diabetes case when we stratified the subjects according to blood glucose. In the normal glucose tolerance population, compared with Q1 (RHR ≤ 71), the risk of albuminuria in the subjects belong to Q3 (79 ≤ RHR ≤ 86) and Q4 (87 ≤ RHR) increased by 13% (OR: 1.13) and 19% (OR: 1.19), respectively, while in the impaired glucose regulation or diabetes mellitus population, the relationship disappeared. The hypoglycemic therapy and the course of diabetes may cause the difference between two studies.

There was also a study extending the previous findings to a population with cardiovascular disease, showing that heart rate is associated with a renal disease risk and suggesting that a reduction in heart rate might be protective for kidney function [21]. In this study, we included the general population (excluding the subject who was diagnosed with cardiovascular disease, kidney disease, tumor history, and related drug application), stratifying the population according to blood pressure. Results showed that in the nonhypertension group, subjects in the Q3 (78 ≤ RHR ≤ 86) quartile and the Q4 (87 ≤ RHR) quartile tend to have a higher UACR; ORs were 1.11 and 1.22, respectively; in the hypertension group, the relationship was of no significance. Perhaps, the effect of hypertension on UACR covered the association between RHR and UACR.

Our study shows that hypertension (OR: 1.66) and diabetes (OR: 1.52) are more significant than RHR (the highest group compared to the lowest group, OR: 1.17) related to UACR. In the general population, the positive relationship between RHR and UACR exists, while in the new diagnostic hypertension and diabetes group, it disappeared. In combination with previous results, we may guess that after effective hypoglycemic and antihypertensive therapy, the relationship between RHR and UACR will reoccur, but it remains further clinical trials. Anyhow, RHR may be a marker of UACR, which reflects early renal impairment.

Several mechanisms may underlie these effects such as progression of atherosclerosis, and thus nephrosclerosis, due to changes in endothelial oxidative stress, which is sensitive to RHR reduction [16, 22]. Furthermore, RHR is associated with increased aortic stiffness, which in turn is linked to cardiovascular disease outcomes such as stroke, coronary events, and heart failure [23–26]. An increased RHR appears to be associated with microvascular disease in general and also with complications of renal failure [27, 28]. In addition, several studies show that an increased RHR can not only increase tensile stress, causing endothelial cell injury, but also increase the permeability of endothelial cells to circulating inflammatory mediators, thus mediated the progression of microalbuminuria [22, 29]. In the kidney, myogenic autoregulation, that is, vasoconstriction of the afferent arteriole in response to high perfusion pressures to prevent hyperperfusion, is impaired in hypertension, ageing, and diabetes, making the kidney more prone to pulsatile stress [30–34]. To date, direct experimental data on heart rate or studies providing information on the relevance of RHR in humans have been lacking. However, it has been reported that increased RHR could also be a sign of increased sympathetic activation [35] and increased sympathetic activation has been observed in patients with renal failure [36]. Besides being a generator of sympathetic activity, the kidney also acts as recipient of efferent signals. Increased renal sympathetic activity leads to a cascade of actions: by stimulating the release of renin by the juxtaglomerular cells, angiotensin II is produced. Angiotensin II directly causes vasoconstriction; renal blood flow and glomerular filtration rate decrease by renal vasoconstriction. This is further amplified by direct activation of the RAS by kidney injury. Increases in renal sympathetic nerve activity also directly increase renal tubular sodium reabsorption [37].

Participants in our study belonged to 8 communities of mainland China, guaranteeing representative results. Compared to other studies, our participants included diverse blood pressure and blood glucose (after excluding the effects of related drugs). Therefore, we fully adjusted confounding such as gender, age, BMI, WC, liver function, and serum lipid. However, several deficiencies exist in this study. First, protein content intake in the previous day and the interval between last meal and sleep were not available; second, UACR levels were determined by a single measurement, but detection methods of the 8 centers were different; third, this is a cross-sectional study; we cannot get a cause-and-effect relationship. Its specific mechanism needs to be explored and confirmed by further basic experiments, but the conclusion still has a certain clinical sense in some aspect.

## 5. Conclusion

Our study reported the association between RHR and UACR in general population. For the population with nonhypertension and normal glucose tolerance, higher RHR is associated with higher UACR. As for the population with hypertension and diabetes, the positive relationship disappeared. After adjusting blood pressure and blood glucose, people with higher RHR tend to have a higher UACR. That indicates RHR may represent an independent risk factor for chronic kidney disease.

## Figures and Tables

**Table 1:** Participants' characteristics according to resting heart rate quartiles.

Variable	RHR quartile (beat/min)				*P* value
	Q1 (≤71)	Q2 (72-78)	Q3 (79-86)	Q4 (≥87)	
Gender	8185	8658	8386	7658	
Male	2890 (35.3)	2501 (28.9)	2387 (28.5)	2280 (29.7)	
Female	5295 (64.7)	6157 (71.1)	5999 (71.5)	5376 (70.3)	
Age (year)	57.1 ± 8.6	56.4 ± 8.8	56.1 ± 8.9	56.5 ± 9.4	
BMI (kg/m^2^)	24.2 ± 3.5	24.3 ± 3.6	24.2 ± 3.5	24.2 ± 3.8	0.337
WC (cm)	84.7 ± 9.8	84.6 ± 9.8	84.4 ± 9.9	84.3 ± 10.4	0.075
HC (cm)	96.2 ± 7.6	96.2 ± 7.9	95.9 ± 7.7	95.8 ± 8.1	0.001
WC/HC					0.656
Smoking					<0.001
Current	1180 (14.4)	1002 (11.6)	967 (11.5)	873 (11.4)	
Former	464 (5.7)	361 (4.2)	352 (4.2)	327 (4.3)	
Never	6541 (79.9)	7295 (84.3)	7067 (84.3)	6456 (84.3)	
Alcohol drinking					<0.001
Current	660 (8.1)	558 (6.4)	577 (6.9)	512 (6.7)	
Former	183 (2.2)	147 (1.7)	150 (1.8)	127 (1.7)	
Never	7342 (89.7)	7953 (91.9)	7659 (91.3)	7017 (91.5)	
SBP (mmHg)	127 ± 19	127 ± 19	127 ± 19	131 ± 20	<0.001
DBP (mmHg)	74 ± 10	75 ± 10	77 ± 10	80 ± 11	<0.001
FPG (mmol/L)	5.53 ± 1.06	5.60 ± 1.16	5.72 ± 1.35	5.95 ± 1.60	<0.001
PPG (mmol/L)	7.28 ± 2.86	7.51 ± 2.85	7.89 ± 3.31	8.42 ± 3.72	<0.001
HbA1c (%)	5.85 ± 0.67	5.87 ± 0.71	5.92 ± 0.85	5.98 ± 1.01	<0.001
Insulin (*μ*U/mL)	7.28 ± 6.37	7.76 ± 4.13	8.06 ± 5.23	8.70 ± 4.94	<0.001
TC (mmol/L)	4.89 ± 1.14	4.95 ± 1.18	5.04 ± 1.19	5.09 ± 1.25	<0.001
TG (mmol/L)	1.44 ± 1.01	1.51 ± 1.07	1.61 ± 1.20	1.69 ± 1.29	<0.001
HDL-c (mmol/L)	1.31 ± 0.35	1.32 ± 0.35	1.33 ± 0.36	1.33 ± 0.36	0.079
LDL-c (mmol/L)	2.88 ± 0.88	2.91 ± 0.91	2.95 ± 0.91	2.99 ± 0.95	<0.001
ALT (mmol/L)	17.1 ± 11.8	17.4 ± 13.3	17.6 ± 13.4	18.6 ± 15.3	<0.001
AST (mmol/L)	21.2 ± 9.2	21.3 ± 11.7	21.4 ± 10.6	22.4 ± 13.5	<0.001
GGT (mmol/L)	25.8 ± 26.4	26.2 ± 30.2	28.1 ± 31.8	32.6 ± 51.6	<0.001
Cr (*μ*mol/L)	67.6 ± 18.8	65.8 ± 14.0	66.3 ± 14.2	67.3 ± 15.0	<0.001
eGFR (mL/min/1.73 m^2^)	96.5 ± 20.8	97.7 ± 21.3	96.7 ± 20.2	95.9 ± 21.9	<0.001
UACR					<0.001
≤25%	2273 (27.8)	2263 (26.1)	2045 (24.4)	1647 (21.5)	
25%-50%	2067 (25.3)	2176 (25.1)	2057 (24.5)	1916 (25.0)	
50%-75%	2008 (24.5)	2129 (24.6)	2134 (25.4)	1947 (25.4)	
>75%	1837 (22.4)	2090 (24.1)	2120 (25.6)	2146 (28.0)	

The values are presented as the means ± the standard deviations or the numbers (percentage).

**Table 2:** Multiple factors related to UACR.

		OR (95% CI)	*P*
HR	Q1 (≤71)		
	Q2 (72-78)	1.05 (0.99, 1.11)	0.101
	Q3 (79-86)	1.13 (1.07, 1.20)	<0.001
	Q4 (≥87)	1.17 (1.10, 1.24)	<0.001

Gender	Man	0.48 (0.45, 0.50)	<0.001
	Woman		

Blood pressure	Hypertension	1.66 (1.59, 1.75)	<0.001
	Nonhypertension		

Blood glucose	DM	1.52 (1.43, 1.63)	<0.001
	IGR	1.06 (1.01, 1.12)	0.012
	NGT		

Age		1.03 (1.02, 1.03)	<0.001

WC		1.50 (1.13, 2.00)	0.005

TG		1.05 (1.03, 1.07)	<0.001

Cr		0.99 (0.99, 1.00)	<0.001

ALT		1.00 (0.99, 1.00)	0.002

AST		1.00 (1.00, 1.01)	0.006

GGT		1.00 (1.00, 1.00)	<0.001

**Table 3:** Relationship between RHR and UACR in total participants.

	Q1	Q2	*P*	Q3	*P*	Q4	*P*
	Reference	OR		OR		OR	
Model 1	1	1.08 (1.03, 1.15)	0.003	1.19 (1.12, 1.25)	<0.001	1.34 (1.27, 1.42)	<0.001
Model 2	1	1.07 (1.01, 1.13)	0.015	1.18 (1.12, 1.25)	<0.001	1.33 (1.26, 1.41)	<0.001
Model 3	1	1.05 (1.00, 1.12)	0.065	1.16 (1.10, 1.23)	<0.001	1.27 (1.20, 1.35)	<0.001
Model 4	1	1.05 (0.99, 1.11)	0.101	1.13 (1.07, 1.20)	<0.001	1.17 (1.10, 1.24)	<0.001

Model 1: unadjusted. Model 2: adjusted for gender, age, BMI, WC, smoking, and alcohol drinking. Model 3: further adjusted for ALT, AST, GGT, TC, TG, HDL-c, and LDL-c. Model 4: further adjusted for SBP, DBP, FPG, and PPG.

**Table 4:** Relationship between RHR and UACR at diverse BP and BG levels.

	Variable	Q1	Q2	*P*	Q3	*P*	Q4	*P*
		OR	OR		OR		OR	
BP	Hypertension	1	1.08 (0.96, 1.21)	0.197	1.20 (1.07, 1.34)	0.002	1.09(0.98, 1.22)	0.129
	Nonhypertension	1	1.04 (0.97, 1.11)	0.281	1.11 (1.04, 1.18)	0.003	1.22 (1.13, 1.31)	<0.001

BG	DM	1	1.04 (0.88, 1.24)	0.631	1.22 (1.03, 1.44)	0.021	1.16 (0.98, 1.36)	0.077
	IGR	1	0.99 (0.88, 1.11)	0.846	1.09 (0.98, 1.22)	0.123	1.14 (1.02, 1.28)	0.020
	NGT	1	1.07 (1.00, 1.15)	0.052	1.13 (1.05, 1.21)	0.002	1.19 (1.10, 1.29)	<0.001

BP: blood pressure; NBP: normal blood pressure; BG: blood glucose; NGT: normal glucose tolerance; IGR: impaired glucose regulation; DM: diabetes mellitus.

## Data Availability

All data used to support the findings of this study are available from the corresponding author upon request.
